# Effects of Two Fractions of *Swietenia macrophylla* and Catechin on Muscle Damage Induced by *Bothrops* Venom and PLA_2_

**DOI:** 10.3390/toxins11010040

**Published:** 2019-01-14

**Authors:** Silvia Posada Arias, Berardo de Jesús Rodríguez, Tatiana Lobo-Echeverri, Raphael Shezaro Ramos, Stephen Hyslop, Vitelbina Núñez Rangel

**Affiliations:** 1 Faculty of Administrative and Agricultural Sciences, Corporación Universitaria Lasallista, Caldas 055440, Colombia; 2 Faculty of Agrarian Sciences, Universidad de Antioquia, Medellín 050036, Colombia; berardo.rodriguez@udea.edu.co; 3 Faculty of Sciences, Universidad Nacional de Colombia, Medellín 050034, Colombia; tloboech@unal.edu.co; 4 Department of Pharmacology, Faculty of Medical Sciences, State University of Campinas (UNICAMP), Campinas, SP 13083-887, Brazil; raphaelschezaroramos@gmail.com (R.S.R.); hyslop@fcm.unicamp.br (S.H.); 5 Microbiology School, Universidad de Antioquia, Medellín 050010, Colombia; vitelbina.nunez@gmail.com

**Keywords:** *Bothrops asper*, *Bothrops marmoratus*, catechin, myonecrosis, plant extracts, toxins

## Abstract

Plant natural products can attenuate the myonecrosis caused by *Bothrops* snake venom and their phospholipases A_2_ (PLA_2_). In this study, we evaluated the effects of two fractions (F4 and F6) from *Swietenia macrophylla* and purified catechin on the muscle damage caused by a myotoxic PLA_2_ from Colombian *Bothrops asper* venom (BaColPLA_2_) in mice and by *Bothrops marmoratus* venom from Brazil in mouse phrenic nerve-diaphragm muscle (PND) preparations in vitro. Male mice were injected with PLA_2_ (50 µg) in the absence or presence of F4, F6, and catechin, in the gastrocnemius muscle and then killed 3, 7, 14, and 28 h later for histopathological analysis of myonecrosis, leukocyte infiltration, and the presence of collagen. Fractions F4 and F6 (500 µg) and catechin (90 µg) significantly reduced the extent of necrosis at all-time intervals. These two fractions and catechin also attenuated the leukocyte infiltration on day 3, as did catechin on day 14. There was medium-to-moderate collagen deposition in all groups up to day 7, but greater deposition on days 14 and 28 in the presence of F6 and catechin. *Bothrops marmoratus* venom (100 µg/mL) caused slight (~25%) muscle facilitation after 10 min and weak neuromuscular blockade (~64% decrease in contractile activity after a 120-min incubation). Pre-incubation of venom with F4 or F6 abolished the facilitation, whereas catechin, which was itself facilitatory, did not. All three fractions attenuated the venom-induced decrease in muscle contractions. These findings indicate that fractions and catechin from *S. macrophylla* can reduce the muscle damage caused by *Bothrops* venom and PLA_2_. These fractions or their components could be useful for treating venom-induced local damage.

## 1. Introduction

Snakebite is a major public health problem in tropical regions of the world, with ~5.5 million bites/year, of which around 400,000 lead to amputation and 20,000–125,000 result in death [1,2,3]. In Colombia, data from the National Vigilance System (SIVIGILA) indicate that 4120 snakebites were reported in 2017, with an average of ~95 bites/week and a national incidence of 10.8 cases/100,000 inhabitants [4].

The genus *Bothrops* (lancehead pit vipers) is responsible for most venomous snakebites in South America [5,6], including Colombia [7]. Myotoxicity is an important local effect of envenomation by *Bothrops* species and is mediated primarily by venom phospholipase A_2_ (PLA_2_) myotoxins that cause extensive damage to skeletal muscle [8]. These myotoxins also produce pronounced edema that can increase the intra-compartmental pressure and compromise the blood flow, which leads to ischemia and necrosis [9]. The combined actions of ischemia and direct muscle damage contribute to the muscle necrosis associated with bites by *Bothrops* spp. [10].

Muscle regeneration subsequent to myonecrosis results in partial to complete structural and functional recuperation, depending on the severity of envenomation [11]. For regeneration to be successful, there must be adequate blood flow, leukocyte infiltration, innervation of the regenerated cells, and the basal lamina around the necrotic muscular fibers must remain intact. A lack of any of these basic requirements will result in poor regeneration [12].

Anti-venoms are very efficient in neutralizing the systemic effects associated with envenomation, but clinical and experimental evidence shows that local effects such as pain, edema, and mytotoxicity are poorly neutralized [10,13,14,15,16,17]. This poor neutralization reflects a combination of the rapid action of the toxins at the bite site, the delay in anti-venom administration, the formation of venom/anti-venom complexes, and the overall kinetics of the venom and anti-venom [16,18,19].

Plant extracts and products constitute a rich source of pharmacologically active compounds, some of which have been shown to inhibit the activity of snake venoms and purified toxins [20,21,22,23,24,25]. This inhibitory activity has been attributed to components such as flavonoids, coumarins, and other polyphenolic metabolites widely distributed in different families of plants [26,27,28,29,30]. Flavonoids such as quercetin (and derivatives), kaempferol, and myricetin [31,32,33,34,35] attenuate or inhibit the local effects (edema, inflammation, hemorrhage, and necrosis) of *Bothrops* snake venoms and selected toxins in experimental animals, either by direct interaction with the venom components or through their antioxidant activities. Catechin (and derivatives), which is a flavonoid with a wide distribution in vascular plants especially in cocoa and tea, also attenuates the local effects of these venoms and their toxins, e.g., gallocatechin inhibits the myotoxicity of BnPLA_2_, a Lys49 PLA_2_ from *Bothrops neuwiedii* venom [36]. However, catechin appears to have limited activity toward venom hyaluronidases [37].

*Swietenia macrophylla* King (Meliaceae) is a medicinal plant used by indigenous people in tropical and subtropical regions around the world, and a variety of activities (antimicrobial, antiinflammatory, antioxidant, antimutagenic, antitumoral, antidiabetic, vasorelaxant, and antihypertensive properties) have been attributed to this species [38,39]. Almost all plant parts are used in traditional medicine for the treatment of various human illnesses [40]. Recent work in vitro has shown that an extract of *S. macrophylla* leaves inhibits the PLA_2_ activity and cytotoxicity of Colombian *Bothrops asper* venom and a PLA_2_-rich fraction of this venom [24,41].

Studies in vitro have shown that an extract of *S. macrohpylla* King inhibits the PLA_2_ activity of *B. asper* venom and a PLA_2_ isolated from this venom [41,42]. In this work, we examined the ability of two fractions of an *S. macrophylla* leaf extract and of catechin (an abundant component in these fractions) to attenuate the myonecrosis caused by a PLA_2_ from Colombian *B. asper* venom in mouse gastrocnemius muscle and to prevent the neuromuscular action of Brazilian *B. marmoratus* venom in mouse isolated phrenic nerve-diaphragm preparations.

## 2. Results

### 2.1. PLA_2_-Induced Necrosis and Its Neutralization by Fractions F4 and F6 and Catechin

**Figure 1:**
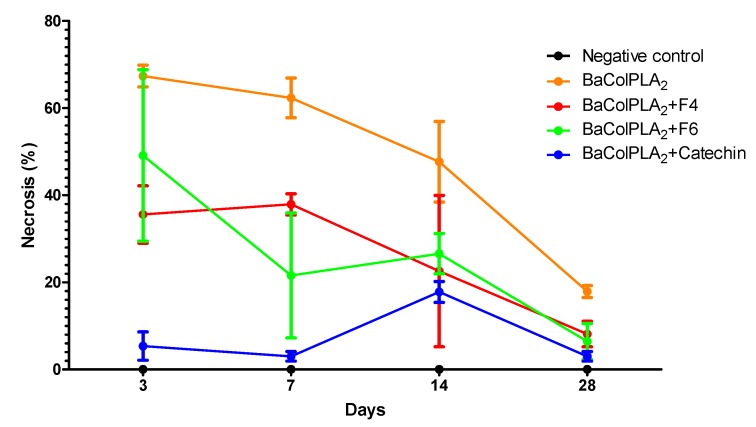
Necrosis caused by BaColPLA_2_ and its inhibition by *S. macrophylla* compounds in mouse gastrocnemius muscle. Mice were injected i.m. with BaColPLA_2_ (50 μg/100μL). Necrosis was expressed as a percentage of the total number of fibers counted. BaColPLA_2_: PLA_2_ from Colombian *B. asper* venom. F4 and F6: fractions 4 and 6 of *S. macrophylla*. Negative control: 0.9% NaCl solution (Note: the controls for F4, F6 and catechin are not shown as they were not significantly different from those for saline.) The points are the mean ± SD (*n* = 4).

Figure 1 shows the extent of muscle necrosis at different intervals after the i.m., injection of BaColPLA_2_ (50 μg). Maximum necrosis (67.3 ± 2.5% of fibers affected) was seen three days post-injection and involved extensive vacuolization and necrosis of the sarcoplasm. Thereafter, there was a progressive decrease in necrosis. However, ~18% of the fibers still showed damage after 28 days. None of the negative control groups (0.9% saline, F4, F6 or catechin) showed necrosis. Inoculation of BaColPLA_2_ with F4, F6, and catechin significantly decreased the necrosis, with catechin being the most effective in preventing muscle damage, particularly in the first seven days post-injection (Figure 1 and Table 1).

### 2.2. Infiltration of Inflammatory Cells

**Figure 2:**
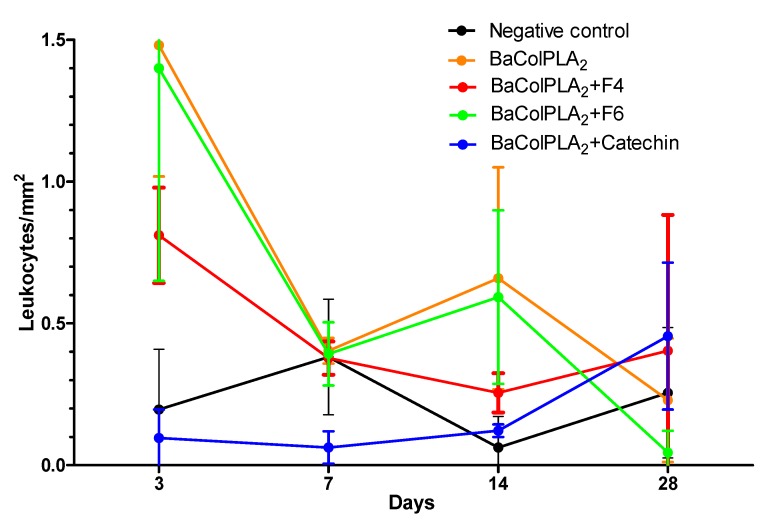
Leukocytic infiltration in mouse gastrocnemius muscle after injection of PLA_2_ (50 µg/100 µL) or PLA_2_+F4, F6, or catechin. BaColPLA_2_: PLA_2_ from Colombian *B. asper* venom.Cat: (+)-catechin. F4 and F6: fractions 4 and 6, respectively, of *S. macrophylla*. The points are the mean ± SD (*n* = 4).

Figure 2 shows the profile of leukocytic infiltration at various times after the i.m. injection of BaColPLA_2_ and Table 2 shows the statistical comparisons for the leukocytic infiltration for each treatment and day. The negative controls (saline solution, F4, F6, and catechin) showed low leukocytic infiltration compared to muscle injected with BaColPLA_2_ alone or BaColPLA_2_+F4, F6, or catechin. In all cases, the most significant differences were observed on day 3. On day 14, significant differences were observed only between muscle injected with BaColPLA_2_ alone and those injected with saline, F4, and BaColPLA_2_+catechin. There were no significant differences among the groups on days 7 and 28.

**Figure 3:**
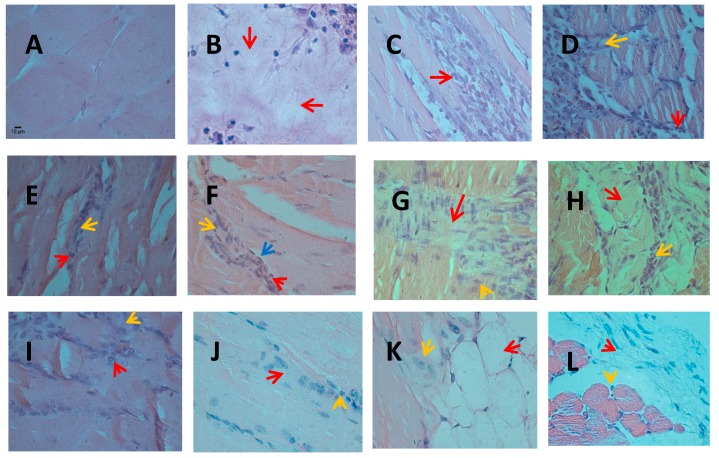
Representative photomicrographs of mouse gastrocnemius muscle with different treatments. (**A**) Negative control (a similar absence of alterations was seen with F4, F6, or catechin alone, not shown). (**B**) Muscle inoculated with BaColPLA_2_ on day 3. Arrow indicates diffuse muscle fiber damage. (**C**) Muscle inoculated with BaColPLA_2_ on day 7. Arrows indicate moderate multifocal leukocytic infiltration among the muscle fibers. (**D**) Appearance of muscle inoculated with BaColPLA_2_ pre-incubated with F4 or F6 on day 3. The red arrow indicates leukocyte infiltration among necrotic muscle fibers and the yellow arrow indicates hypertrophic fibroblasts. (**E**) Muscle inoculated with BaColPLA_2_ pre-incubated with catechin on day 3. The red arrow indicates slight infiltration of neutrophils and the yellow arrow indicates slight fibrosis. (**F**) Muscle inoculated with BaColPLA_2_ pre-incubated with F4 on day 7. The red arrow indicates infiltration of neutrophils, the yellow arrow indicates discrete fibrosis and the blue arrow indicates a satellite cell. (**G**) Muscle inoculated with BaColPLA_2_ pre-incubated with catechin on day 7. The red arrow indicates discrete focal fibrosis and the yellow arrow indicates a mild neutrophil infiltrate. (**H**) Muscle inoculated with BaColPLA_2_ on day 14. The red arrow indicates small foci of myonecrosis and the yellow arrow indicates a moderate number of neutrophils. (**I**) Appearance of muscle inoculated with BaColPLA_2_ pre-incubated with F4 or F6 on day 14. The red arrow indicates moderate multifocal infiltrate of neutrophils and the yellow arrow indicates discrete focal fibrosis. (**J**) Muscle inoculated with BaColPLA_2_ pre-incubated with catechin on day 14. The red arrow indicates discrete focal fibrosis and the yellow arrow indicates a slight infiltration of neutrophils. (**K**) Muscle inoculated with BaColPLA_2_ on day 28. The red arrow indicates focal infiltration of the adipose tissue. The yellow arrow indicates discrete neutrophil infiltration. (**L**) Muscle inoculated with BaColPLA_2_ pre-incubated with catechin on day 7. The red arrow indicates occasional fibrosis and the yellow arrow indicates a few neutrophils in the surrounding adipose tissue. Scale bar represents 10 µm (all panels). Hematoxylin &Eosin stain. 100×.

There were no significant histopathological alterations in the control groups (saline solution, F6, F4, and catechin) at the various intervals examined (Figure 3A).

On day 3, the group inoculated with BaColPLA_2_ showed severe, diffuse muscle fiber damage with vacuolization and necrosis of the sarcoplasm (Figure 3B). The findings on day 7 were similar tothoseon day 3. In mice injected with BaColPLA_2_ alone, there was active chronic myositis characterized by a moderate multifocal leukocytic infiltration that consisted primarily of neutrophils and a few macrophages distributed among the muscle fibers and in surrounding adipose tissue (Figure 3C). In mice injected with BaColPLA_2_ and F4 or F6, there was a leukocyte infiltration that consisted of neutrophils, lymphocytes, macrophages, and eosinophils distributed among necrotic muscle fibers, abundant satellite myogenic cells, and hypertrophic fibroblasts. There was also slight focal edema and moderate multifocal vascular congestion (Figure 3D). Mice injected with BaColPLA_2_+catechin showed only exudative myositis with a slight infiltration of neutrophils and slight fibrosis (Figure 3E).

In muscle injected with BaColPLA_2_+F6, there was evident repair and focal fibrosis that extended to the surrounding adipose tissue. There was also a discrete presence of neutrophils. In muscle injected with BaColPLA_2_+F4, chronic active myositis was observed along with repair in which a focal proliferation of myogenic satellite cells was associated with an infiltration of neutrophils and discrete fibrosis (Figure 3F). There was an occasional neutrophilic infiltration in the surrounding adipose tissue. In mice inoculated with BaColPLA_2_+catechin, a repair process accompanied by discrete focal fibrosis, a mild neutrophil infiltrate and associated vascular congestion was observed (Figure 3G).

On day 14, there were significant differences between muscle injected with BaColPLA_2_ alone and those injected with BaColPLA_2_+F4, BaColPLA_2_+F6, and BaColPLA_2_+catechin, but there were no differences among the latter three groups (Table 2). In muscle injected with BaColPLA_2_, there were small foci of myonecrosis infiltrated by a moderate number of neutrophils and macrophages, along with myogenic satellite cells and hypertrophic fibroblasts (Figure 3H).There was moderate vascular congestion and a moderate neutrophilic infiltration in the surrounding adipose tissue. In muscle injected with BaColPLA_2_+F4 and BaColPLA_2_+F6, there was a moderate multifocal infiltrate of neutrophils and a few lymphocytes distributed in adipose and connective tissues and among muscle fibers. There was also discrete focal fibrosis and moderate vascular congestion (Figure 3I). In muscle injected with BaColPLA_2_+catechin, there was tissue repair with discrete focal fibrosis, which is a slight infiltration of neutrophils and vascular congestion (Figure 3J).

On day 28, there were significant differences in the profile of neutrophil infiltration among muscles injected with BaColPLA_2_ alone and those injected with BaColPLA_2_+F4, F6 or catechin (Table 2), but there were no differences among the latter three groups. In muscle injected with BaColPLA_2_, there was focal infiltration of the adipose tissue, slight fibrosis, and discrete neutrophil infiltration, as well as moderate vascular congestion (Figure 3K). In muscle injected with BaColPLA_2_+F4, there was moderate multifocal infiltration of neutrophils and focal proliferation of hypertrophic fibroblasts in the adjacent muscle tissue. There was also slight vascular congestion. In muscle injected with BaColPLA_2_+F6, there was discrete focal fibrosis, while in muscle injected with BaColPLA_2_+catechin there was occasional fibrosis and a few neutrophils in the surrounding adipose tissue (Figure 3L).

### 2.3. Fibrosis

**Figure 4:**
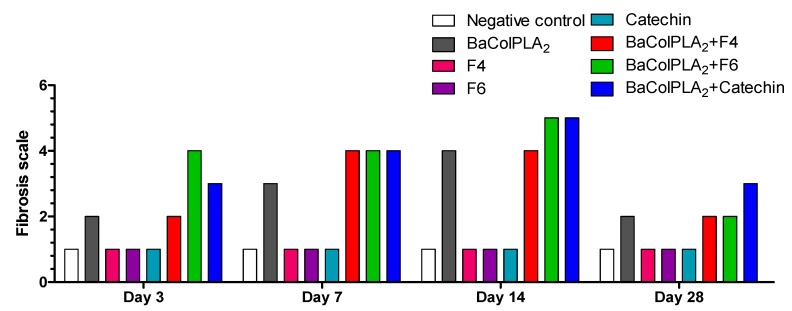
Fibrosis based on the presence of collagenous fibers assessed 3, 7, 14, and 28 days after the injection of PLA_2_ (50 µg/100µL) or PLA_2_+F4, F6, or catechin (all in a fixed volume of 100 µL). BaColPLA_2_: PLA_2_ from Colombian *B. asper* venom. Cat: (+)-catechin. F4 and F6: fractions 4 and 6, respectively, of *S. macrophylla.* Negative control—saline solution.

**Figure 5:**
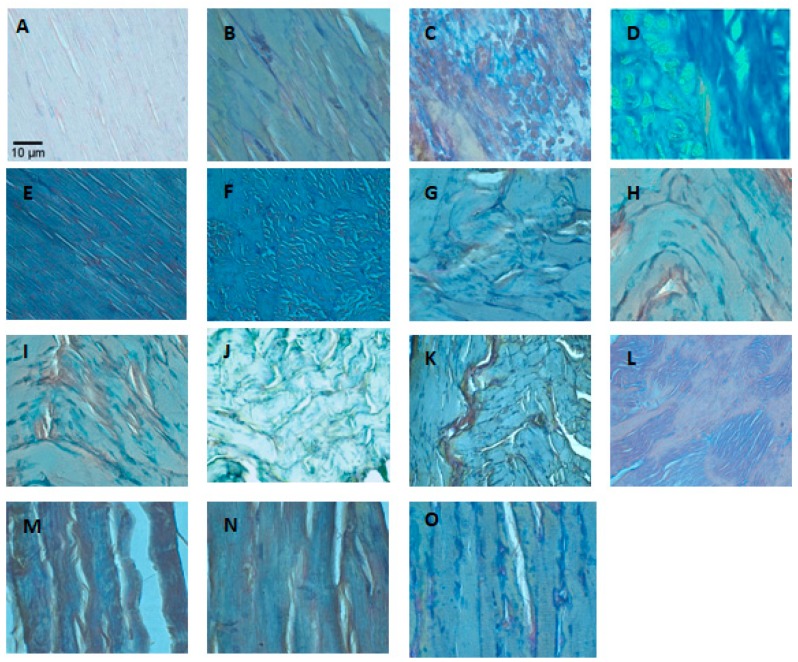
Representative photomicrographs of fibrosis in control and PLA_2_-treated mouse gastrocnemius. (**A**) Absence of collagen in all negative controls at all intervals evaluated. (**B**) BaColPLA_2_ day 3. (**C**) BaColPLA_2_+F4 day 3. (**D**) BaColPLA_2_+F6 day 3. (**E**) BaColPLA_2_+Catechin day 3. (**F**) BaColPLA_2_ day 7. (**G**) BaColPLA_2_+F4 day 7. (**H**) BaColPLA_2_+F6 day 7. (**I**) BaColPLA_2_+Catechin day 7. (**J**) BaColPLA_2_+F6 day 14. (**K**) BaColPLA_2_+Catechin day 14. (**L**) BaColPLA_2_ day 28. (**M**) BaColPLA_2_+F4 day 28. (**N**) BaColPLA_2_+F6 day 28. (**O**) BaColPLA_2_+Catechin day 28. Scale bar represents 10 µm (all panels). Masson’s trichrome stain. 100×.

Figure 4 shows the extent of fibrosis based on the presence of collagen at various intervals after each treatment and Figure 5 shows representative photomicrographs of each group. On day 3, muscle injected with BaColPLA_2_ alone or BaColPLA_2_+F4 showed a medium level for fibrosis, while muscle injected with BaColPLA_2_+F6 and BaColPLA_2_+catechin showed moderate to severe fibrosis (see Table 3 for classification of the extent of fibrosis). On day 7, muscle injected with BaColPLA_2_ alone showed medium to moderate fibrosis, while the three groups injected with fractions or catechin showed moderate fibrosis. On day 14, muscle injected with BaColPLA_2_+F6 and BaColPLA_2_+catechin showed moderate to severe fibrosis, with 50% to 80% of the sections showing collagenous fibers. The muscle from the other groups showed similar levels of fibrosis to those seen on day 7. By day 28, the level of fibrosis in all groups (except for BaColPLA_2_+F6) had returned to that seen on day 3. There was no collagen deposition in any of the control groups at any of the intervals examined.

### 2.4. Neuromuscular Activity of B. Marmoratus Venom

**Figure 6:**
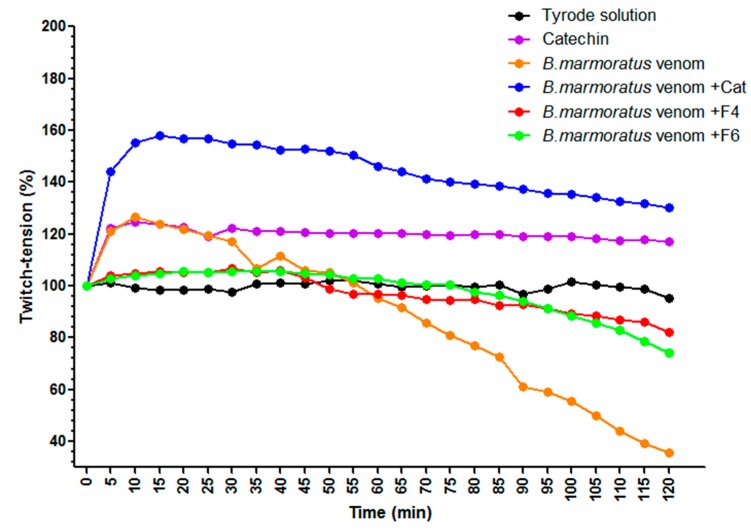
Neuromuscular activity of *B. marmoratus* venom (100 μg/mL) in mouse phrenic nerve-diaphragm preparations. The responses to F4 and F6 were not significantly different from those for Tyrode solution and are not shown in the graph. In contrast, catechin alone caused sustained facilitation of the twitch-tension responses. Note the venom-induced facilitation that was maximal 10 min after addition was followed by attenuation of the twitch-tension responses that were significantly different (*p* < 0.05) from the pre-venom basal values from 85 min onwards. Pre-incubation of venom with F4 and F6 virtually abolished the facilitatory and inhibitory effects of the venom, whereas pre-incubation with catechin resulted in even greater facilitation that decreased progressively throughout the experiment.

**Figure 7:**
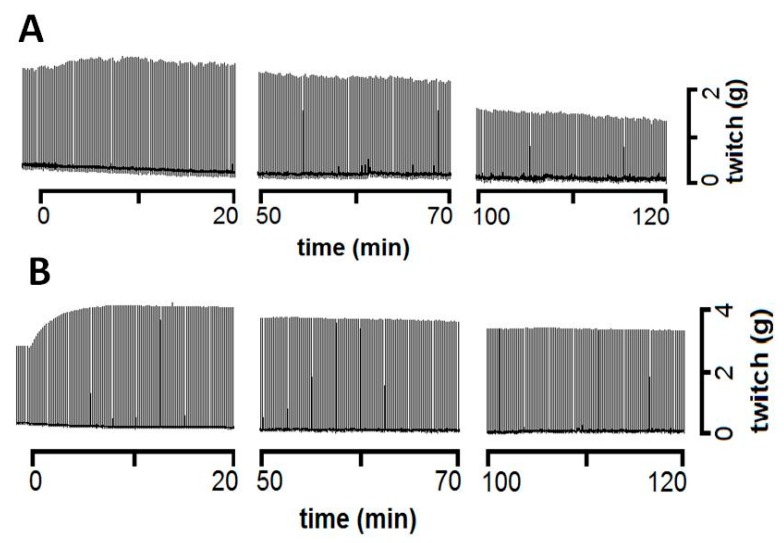
Representative recordings of the twitch-tension responses of mouse phrenic nerve-diaphragm preparations incubated with (**A**) *B. marmoratus* venom (100 µg/mL) and (**B**) *B. marmoratus* venom pre-incubated with 3.1 mM catechin prior to addition to the organ bath. Pre-incubation with catechin exacerbated the facilitation and inhibited the venom-induced decrease in contractile force. The recordings are representative of three experiments in each case.

*Bothrops marmoratus* venom (100 µg/mL) produced initial facilitation of muscle contractile responses (~25% after 10 min, *p* < 0.05 relative to basal pre-venom values) followed by a progressive decrease in twitch-tension that reached 50% after 100 min and ~64% after 120 min. Catechin alone produced a slight facilitation (~25%) of muscle responses that was maintained throughout the 2-h incubation but was not significantly different from the responses in control preparations incubated with Tyrode solution alone. The pre-incubation of venom with catechin resulted in enhanced muscle facilitation that was significantly greater (*p* < 0.001) than the responses to venom or catechin alone throughout the incubation. However, the overall effect appeared to be more additive than synergistic. The pre-incubation of venom with F4 and F6 prevented the venom-induced facilitation and subsequent decrease in twitch tension responses, with the resulting curves resembling those obtained for the negative control after 120 min (Figure 6). Figure 7 shows representative recordings of experiments with venom alone and venom + catechin.

## 3. Discussion

Muscle repair after envenomation by *Bothrops* species, following treatment with anti-venom, is often incomplete and is characterized by a loss of tissue and a functional deficit because of defective tissue regeneration [8,10,43]. However, the relative contribution of venom components (metalloproteinases, PLA_2_, etc.,) to this damage varies and may differ in overall profile and severity when compared with the damage caused by whole venom [11].

In agreement with our previous findings [42], BaColPLA_2_ caused myonecrosis in mouse gastrocnemius muscle that included severe, diffuse fiber damage with vacuolization and necrosis of the sarcoplasm. In our study, we demonstrated that the muscle damage was repaired within 28 days. These results agree with the observations of Hernández et al. [11], who compared the tissue repair after damage caused by a myotoxin, a metalloproteinase and venom from Costa Rican *B. asper*.

Complete proper tissue repair depends on an adequate blood flow, innervation, and persistence of the basal lamina. Since myotoxins damage the vasculature and basal lamina, the subsequent repair has been shown to involve both structural and functional aspects [11,44,45]. Thus, on day 28, there were no marked differences in the damage among muscles of the different groups injected with PLA_2_. However, at earlier intervals (days 7 and 14), treatment with catechin and fractions F4 and F6 of *S. macrophylla* attenuated the necrosis compared to muscle injected with PLA_2_ alone. These results obtained in vivo were in accordance with studies of enzymatic inhibition in vitro. Specifically, Preciado et al. [41] reported that F4 and F6 inhibited the activity of *B. asper* PLA_2_ by 71 ± 8% and 63 ± 4%, respectively, at a PLA_2_:F4 or F6 ratio of 1:10.These authors also used molecular docking to show that (+)-catechin blocked the entrance of the hydrophobic channel of PLA_2_ from South American rattlesnake (*Crotalus durissus terrificus*) venom. In F4, in addition to catechin, palmitic and oleic acids were identified, whereas in F6, palmitic, stearic, and protocatechuic acids were identified [41].

Repair mediated by inflammation was observed in all PLA_2_-treated groups, with a marked leukocytic infiltration in the first days in muscle injected with PLA_2_ alone. Muscle injected with PLA_2_+F4 or F6 showed less leukocytic infiltration that was significantly lower than with PLA_2_ alone on day 3, but not on days 7 and 28. Muscle injected with PLA_2_+catechin had a significantly lower number of leukocytes than the other groups at the same intervals. These findings agreed with the histopathological analysis in which muscle injected with PLA_2_+catechin showed a very low percentage of necrosis.

Pithayanukul and Leanpolchareanchai [29] evaluated the inhibitory effects of tea (*Camellia sinensis*) polyphenols on local tissue damage induced by venom from the snakes *Naja naja kaouthia* and *Calloselasma rhodostoma*. A *C. sinensis* extract inhibited PLA_2_, proteases, hyaluronidase, and L-amino acid oxidase of both venoms in vitro and inhibited their hemorrhagic and dermonecrotic activities in vivo. This inhibitory activity was attributed to the complexation and chelation of venom proteins by phenolic components of the extract. These authors proposed a mechanism by which polyphenols inhibited cyclooxygenase and lipoxygenase, and postulated that the anti-inflammatory activities of the extract may have a synergistic action with its inhibitory activity against the venom enzymes. This combined effect contributed to the prevention of global tissue damage. These same mechanisms are suggested for our results because catechin is a polyphenol.

Ticli et al. [46] evaluated the effect of rosmarinic acid obtained from *Cordia verbenacea* (‘baleeira’, ‘whaler’) and reported its anti-inflammatory and anti-myotoxic activities against the basic PLA_2_s bothropstoxins I and II (BthTX-I and BthTX-II) from *Bothrops jararacussu* snake venom. Rosmarinic acid was modeled in the hydrophobic channel leading to the active site and was shown to remain in this channel by interacting with the hydroxyl group of one of the aromatic rings bound to His48 and the carboxyl group bound to Lys69. This finding reinforced the hypothesis about the general mechanism of action of polyphenols against PLA_2_.

As we observed with the effects on myonecrosis, Cotrim et al. [47] showed that quercetin inhibited the enzymatic activity and some pharmacological activities of venom PLA_2_, including its antibacterial activity, its ability to induce platelet aggregation and myotoxicity. Docking experiments revealed the existence of hydrogen-bonded polar and hydrophobic interactions, which suggested that other flavonoids with similar structures may also bind to PLA_2_. This mechanism was proposed for catechin, which is a major component in the fractions of *S. macrophylla* identified by gas chromatography coupled to mass spectrometry [41].

Snake venom-induced necrosis is accompanied by an inflammatory response in which the tissue is invaded by neutrophils and macrophages [12,48]. In addition to removing necrotic debris, these white cells synthesize cytokines and growth factors that modulate regeneration. The leukocytic infiltrate seen here in muscle injected with PLA_2_ alone agreed with the general findings for local tissue damage by *B. asper* venom [18], i.e., a limited infiltrate at 6 h that increased 24 to 72 h after injection. The cellular infiltrate was observed in necrotic muscle cells and in the interstitial space.

Teixeira et al. [49] examined the role of neutrophils in envenomed mice. Depletion of the number of neutrophils did not affect the amount of tissue damage, but did affect muscle regeneration after myonecrosis induced by a myotoxic PLA_2_. This finding agreed with our results for muscle injected with BaColPLA_2_. In addition, Robertson et al. [50] posited that the depletion of neutrophils may jeopardize the adequate removal of necrotic debris and the recruitment of macrophages that play a key role in muscle regeneration. Zamuner et al. [51] also stated that, despite a prominent infiltration of neutrophils in envenomed tissue and the production of reactive species of oxygen and nitrogen, neutrophils likely played a protective role in the *Bothrops* venom-induced tissue damage because of their role in muscle regeneration.

Fibroblasts and connective tissue are important components of muscle regeneration under normal conditions. As shown here, on day 3, there was a greater deposition of collagen fibers, which indicates fibroblast activity in muscle injected with PLA_2_+F6. However, in all groups, the pattern of fibrosis was similar to the normal repair processes described by Kaariainen et al. [52]. According to these authors, necrotic muscular cells are replaced by a scar of connective tissue formed by type I and II collagen beginning as early as 3 days post-injury. The fibrotic tissue provides early support for the broken myofibers, which agrees with the greater abundance of collagen that we observed between days 7 and 14. However, muscle injected with PLA_2_+F4, F6, or catechin had more collagen than muscle injected with PLA_2_ alone. Since the temporal proliferation of connective tissue elements may serve as an additional support for muscle, nerve, and blood vessel fibers, compounds present in F4 and F6, as well as catechin, may favor the repair process. In this regard, fibroblasts are important in promoting the migration of activated satellite cells to the site of injury and regeneration [12].

Muscle injected with PLA_2_+catechin showed a higher level of collagen than the other groups on day 28. This effect of catechin on collagen production and subsequent fibroblast proliferation could be partly related to the release of growth factors. Tanigawa et al. [53] described a protective effect of catechin against oxidative stress-induced apoptosis in dermal fibroblasts, possibly by inhibiting the phosphorylation of p38 and JNK. Silva Santos et al. [54] described the effect of catechin and epicatechin in attenuating mitochondrial dysfunction and oxidative stress induced by amiodarone in human lung fibroblasts. Catechin, resveratrol, and quercetin can directly or indirectly enhance the expression of proteins called sirtuins. These are mainly protein deacetylases involved in diverse cellular processes and pathways, and vary in cell localization and functions, such as cellular proliferation. SirtuinSIRT1induces the production of fibroblast growth factor 21 [55].

In muscle injected with PLA_2_ alone and with PLA_2_+F4 or F6, the levels of collagen were low on day 28 since excess connective tissue was removed as the muscle was repaired. However, with persistent injury, inflammation dominates due to the infiltration of macrophages [12]. As shown here, by day 28, there was a decrease in leukocytic infiltration and in the abundance of collagen, which is in agreement with other findings [56,57,58,59,60,61].

During muscle repair, the fibroblastic phenotype may switch to an adipocytic phenotype [12]. In the present study, zones infiltrated by adipose tissue in muscle injected with PLA_2_ alone were observed on day 28, but not in the treated groups. This observation suggested that the presence of adipocytes in the former group could contribute to inadequate repair that the fractions and catechin can prevent.

In mouse phrenic nerve-diaphragm preparations, incubation with *B. marmoratus* venom resulted in an initial, transitory facilitatory response followed by a ~64% decrease in contractile activity after 2 h. These alterations were abolished by pre-incubating the venom with F4 and F6, whereas the muscle facilitation seen with catechin alone was even greater in the presence of the venom. Similar results were reported by dos Santos et al. [62], who noted that rosmarinic acid significantly prevented the neuromuscular blockade induced by PrTX-I, a Lys49 PLA_2_ from *Bothrops pirajai* when both were pre-incubated. Rosmarinic acid reduced the muscle damage and neuromuscular blockade by 80% and 90%, respectively.

Overall, the results of this investigation indicate that fractions F4 and F6 of *S. macrophylla* can attenuate snake venom PLA_2_ activity in vitro and reduce the extent of myonecrosis and inflammation and attenuate the neuromuscular damage caused by this toxin. These findings suggest that compounds present in *S. macrophylla* could provide useful leads for developing therapeutic alternatives for treating the local effects of *Bothrops* venoms.

## 4. Materials and Methods

### 4.1. Animals

Male Swiss-Webster mice were supplied by the central Animal House (Sede de Investigación Universitaria—SIU) at Universidad de Antioquia and male BALB/c mice were obtained from the Multidisciplinary Center for Biological Investigation (CEMIB/UNICAMP). The mice were housed 5/cage at 22 ± 1 °C on a 12 h light/dark cycle, with lights on at 6 a.m., and were provided with standard rodent chow (Nuvilab^®^) and water *ad libitum*. The experiments with animals were approved by the relevant Animal Care and Use Committees of the Universidad de Antioquia (license nos. 70 (2011) and 102 (2016) and the State University of Campinas (CEUA/UNICAMP, protocol no. 4707-1/2017, approved 7 December 2017).

### 4.2. Venoms and PLA_2_

*Bothrops asper* venom from 30 specimens collected in the Middle Magdalena region of Antioquia was provided by the serpentarium of the Universidad de Antioquia. A PLA_2_ (BaColPLA_2_) was purified from this venom as previously described [35]. *Bothrops marmoratus* venom was obtained from specimens collected in the Brazilian state of Goiás and was kindly provided by Dr. Nelson Jorge da Silva Jr. (PUC-Goiás, Goiânia, GO, Brazil). In both cases, the venoms were centrifuged, lyophilized, and stored at −70 °C. For use, the PLA_2_ and venom were reconstituted in 0.9% NaCl and kept at 4 °C until required.

### 4.3. Extracts and Compounds of S. macrophylla

Extracts of *S. macrophylla* were obtained using the protocol described by Preciado et al. [41]. Bio-directed fractionation to identify the possible substances responsible for the inhibitory activity of *S. macrophylla* yielded eight fractions of which the most active were F4, F5, and F6, based on their ability to inhibit BaColPLA_2_ and their antioxidant capacity. In the initial screening for myotoxicity, F5 increased the activity of circulating creatine kinase and was, therefore, excluded from this study. Analysis of F4, F5, and F6 showed that (+)-catechin was the most abundant component. Based on these findings, the extracts and compounds evaluated in this study were F4, F6, and (+)-catechin.

### 4.4. Histological Analysis

Groups of four mice were inoculated in the right gastrocnemius muscle with either BaColPLA_2_ (50 µg/100 µL of 0.9% NaCl) or BaColPLA_2_ (50 µg/50 µL 0.9% NaCl) and 30 s later with F4 or F6 (500 µg/50 µL 0.9% NaCl) or catechin (90 µg/50 µL 0.9% NaCl) in a total inoculation volume of 100 µL. We performed cytotoxicity tests with 1:5, 1:10, and 1:20 ratios and it was seen that the 1:10 ratio provided the best protection (data not shown). For this reason, the myotoxicity protocol for the toxin was done using 50 µg [42], and a 10 times higher amount of the fractions (500 µg/50 µL). Catechin accounted for 18% of the fraction weight, as previously reported [41]. For this reason, 90 µg of catechin was used in the assays. Control mice were injected with 0.9% NaCl, F4, F6, or catechin alone (in 100 µL) under the same conditions. At pre-established intervals (days 3, 7, 14, and 28 after injection of the test substances), the mice were killed with CO_2_ (as recommended by the Guide for the Management and Care of Experimental Animals of the Universidad de Antioquia). The gastrocnemius muscle was removed, fixed in 10% buffered formalin, embedded in paraffin, and four sections 4 µm thick were obtained per muscle. The sections were stained with hematoxylin-eosin to assess necrosis and leukocyte infiltration or with Masson’s trichrome to assess fibrosis based on the presence of collagen [63]. The quantification of fibrous tissue was carried out using Image J software (National Institutes of Health, v.1.5 Java, Bethesda, Maryland, United States, 2016). The sections were examined with a Leica DMLB microscope (Meyer Instruments, Houston, TX, USA). Three non-overlapping images were obtained from each section and quantitative analysis was done. The total area was measured (in pixels) and the amount of extracellular matrix stained in blue (with Masson’s trichromic), expressed as apercentage of the total area, was considered indicative of the extent of fibrosis. 

Muscle necrosis was detected based on the presence of hypercontracted fibers, fiber degeneration, and delta lesions. The extent of necrosis was assessed by examining four non-consecutive transversal sections of each muscle and the percentage of necrotic fibers was expressed relative to the total number of fibers counted in each section. The mean percentage of necrosis for the sections examined per mouse represented the extent of necrosis for that mouse. The mean percentage of necrosis for each group was then calculated using the values determined for all mice in the group.

Leukocyte infiltration was evaluated by direct counting of the cells and expressing the number/mm^2^. For this, three non-overlapping images per section from each mouse were examined at 100× magnification for each of the post-injection intervals. A total area of 90 mm^2^ was examined for each muscle. Fibrosis was assessed based on a semi-quantitative analysis of the presence of collagen as indicated in Table 3.

All the samples were processed and analyzed in the Laboratory of Animal Pathology of the Universidad de Antioquia.

### 4.5. Mouse Phrenic Nerve-Diaphragm Preparation

Phrenic nerve-diaphragm preparations obtained from male mice killed with an overdose of isofluorane were mounted under a resting tension of 5 g in a 5 mL organ bath containing aerated (95% O_2_ and 5% CO_2_) Tyrode solution (composition, in mM: NaCl 137, KCl 2.7, CaCl_2_ 1.8, MgCl_2_ 0.49, NaH_2_PO_4_ 0.42, NaHCO_3_ 11.9, and glucose 11.1) at 37 °C [64].Supramaximal stimuli (0.1 Hz and 0.2 ms for indirect stimulation) were delivered from a Grass S88 stimulator (Grass Instrument Co., Quincy, MA, USA) and muscle twitches were recorded using a Model MLT0201 force transducer 5 mg–25 g (Panlabsl, AD Instruments Pty Ltd., Barcelona, Spain) connected to a Power Lab/4SP (Quad Bridge, AD Instruments, Barcelona, Spain). After stabilization for 20 min, *B. marmoratus* venom (100 µg/mL, final concentration in the organ bath) alone or venom pre-incubated with F4, F6, or catechin was added to the preparations and left in contact for 120 min to examine their influence on muscle contractile responses. In the pre-incubation protocols, the venom was incubated with fractions in a venom:fraction ratio of 1:10 or with 3.1 mM catechin for 20 min. Negative controls consisted of fractions F4 and F6 or catechin alone.

### 4.6. Statistical Analysis

Numerical data were expressed as the mean ± SD of the number of experiments indicated in the figure legends. Statistical comparisons between treatments and days were conducted using two-way ANOVA followed by the Bonferroni test, with *p* < 0.05 indicating significance. All data analyses were completed using Prism v.5.01 software for Windows (GraphPad Inc., San Diego, CA, USA).

## Figures and Tables

**Table 1:** The effects of F4, F6, and catechin on PLA_2_-induced necrosis were compared using two-way ANOVA followed by the Bonferroni test, with *p* < 0.05 indicating significance. BaColPLA_2_: PLA_2_ from Colombian *B. asper* venom. Cat: (+)-catechin. F4 and F6: fractions 4 and 6 of *S. macrophylla*, respectively. ns—not significant.

Treatment	Days Post-Injection			
	3	7	14	28
BaColPLA_2_ vs Saline	*p* < 0.001	*p* < 0.001	*p* < 0.001	*p* < 0.001
BaColPLA_2_ vs F4	*p* < 0.001	*p* < 0.001	*p* < 0.001	*p* < 0.001
BaColPLA_2_ vs F6	*p* < 0.001	*p* < 0.001	*p* < 0.001	*p* < 0.001
BaColPLA_2_ vs Cat	*p* < 0.001	*p* < 0.001	*p* < 0.001	*p* < 0.001
BaColPLA_2_ vs BaColPLA_2_+F4	*p* < 0.001	*p* < 0.001	*p* < 0.001	*p* < 0.01
BaColPLA_2_ vs BaColPLA_2_+F6	*p* < 0.001	*p* < 0.001	*p* < 0.001	*p* < 0.001
BaColPLA_2_ vs BaColPLA_2_+Cat	*p* < 0.001	*p* < 0.001	*p* < 0.001	*p* < 0.001
BaColPLA_2_+F4 vs BaColPLA_2_+F6	*p* < 0.001	*p* < 0.001	ns	ns
BaColPLA_2_+F4 vs BaColPLA_2_+Cat	*p* < 0.001	*p* < 0.001	ns	ns
BaColPLA_2_+F6 vs BaColPLA_2_+Cat	*p* < 0.001	*p* < 0.001	*p* < 0.01	ns

**Table 2:** The effects of F4, F6, and catechin on PLA_2_-induced leukocyte infiltration were compared using two-way ANOVA followed by the Bonferroni test, with *p* < 0.05 indicating significance. BaColPLA_2_: PLA_2_ from Colombian *B. asper* venom. Cat: (+)-catechin. F4 and F6: fractions 4 and 6 of *S. macrophylla*, respectively. ns—not significant.

Treatment	Days Post-Injection			
	3	7	14	28
BaColPLA_2_ vs Saline	*p* < 0.001	ns	*p* < 0.05	ns
BaColPLA_2_ vs F4	*p* < 0.001	ns	*p* < 0.05	ns
BaColPLA_2_ vs F6	*p* < 0.001	ns	ns	ns
BaColPLA_2_ vs Cat	*p* < 0.001	ns	ns	ns
BaColPLA_2_ vs BaColPLA_2_+F4	*p* < 0.01	ns	ns	ns
BaColPLA_2_ vs BaColPLA_2_+F6	*p* < 0.01	ns	ns	ns
BaColPLA_2_ vs BaColPLA_2_+Cat	*p* < 0.001	ns	*p* < 0.05	ns
BaColPLA_2_+F4 vsBaColPLA_2_+F6	*p* < 0.05	ns	ns	ns
BaColPLA_2_+F4 vs BaColPLA_2_+Cat	*p* < 0.01	ns	ns	ns
BaColPLA_2_+F6 vs BaColPLA_2_+Cat	*p* < 0.001	ns	ns	ns

**Table 3:** Used to assess the extent of fibrosis based on the presence of collagen (adapted from Henao-Duque et al. [63]).

Score	Extent of Fibrosis	Interpretation of Findings
1	Absent	No collagen deposition
2	Focal, 15% of tissue affected	Medium fibrosis
3	Focal, 15–35% of tissue affected	Medium to moderate fibrosis
4	Multifocal, 35–50% of tissue affected	Moderate fibrosis
5	Multifocal, 50–85% of tissue affected	Moderate to severe fibrosis
6	Extensive, diffuse, 85–100% of tissue affected	Severe fibrosis
